# Ameliorating Effects of Graphene Oxide on Cadmium Accumulation and Eco-Physiological Characteristics in a Greening Hyperaccumulator (*Lonicera japonica* Thunb.)

**DOI:** 10.3390/plants13010019

**Published:** 2023-12-20

**Authors:** Zhouli Liu, Qingxuan Lu, Yi Zhao, Jianbing Wei, Miao Liu, Xiangbo Duan, Maosen Lin

**Affiliations:** 1 College of Life Science and Engineering, Shenyang University, Shenyang 110044, China; lqx0812@syu.edu.cn (Q.L.); oliver1208@sina.com (J.W.); duanxiangbo@aliyun.com (X.D.); 2 Institute of Carbon Neutrality Technology and Policy, Shenyang University, Shenyang 110044, China; 3 Northeast Geological S&T Innovation Center of China Geological Survey, Shenyang 110000, China; 4 Key Laboratory of Black Soil Evolution and Ecological Effect, Ministry of Natural Resources, Shenyang 110000, China; 5 School of Chemistry and Environmental Engineering, Liaoning University of Technology, Jinzhou 121001, China; 6 Institute of Applied Ecology, Chinese Academy of Sciences, Shenyang 110016, China; lium@iae.ac.cn; 7 College of Water Conservancy, Shenyang Agricultural University, Shenyang 110161, China

**Keywords:** graphene oxide, cadmium, greening plants, hyperaccumulator, response

## Abstract

Graphene oxide (GO), as a novel carbon-based nanomaterial (CBN), has been widely applied to every respect of social life due to its unique composite properties. The widespread use of GO inevitably promotes its interaction with heavy metal cadmium (Cd), and influences its functional behavior. However, little information is available on the effects of GO on greening hyperaccumulators under co-occurring Cd. In this study, we chose a typical greening hyperaccumulator (*Lonicera japonica* Thunb.) to show the effect of GO on Cd accumulation, growth, net photosynthesis rate (P_n_), carbon sequestration and oxygen release functions of the plant under Cd stress. The different GO-Cd treatments were set up by (0, 10, 50 and 100 mg L^−1^) GO and (0, 5 and 25 mg L^−1^) Cd in solution culture. The maximum rate of Cd accumulation in the roots and shoots of the plant were increased by 10 mg L^−1^ GO (exposed to 5 mg L^−1^ Cd), indicating that low-concentration GO (10 mg L^−1^) combined with low-concentration Cd (5 mg L^−1^) might stimulate the absorption of Cd by *L*. *japonica*. Under GO treatments without Cd, the dry weight of root and shoot biomass, P_n_ value, carbon sequestration per unit leaf area and oxygen release per unit leaf area all increased in various degrees, especially under 10 mg L^−1^ GO, were 20.67%, 12.04%, 35% and 28.73% higher than the control. Under GO-Cd treatments, it is observed that the cooperation of low-concentration GO (10 mg L^−1^) and low-concentration Cd (5 mg L^−1^) could significantly stimulate Cd accumulation, growth, photosynthesis, carbon sequestration and oxygen release functions of the plant. These results indicated that suitable concentrations of GO could significantly alleviate the effects of Cd on *L. japonica*, which is helpful for expanding the phytoremediation application of greening hyperaccumulators faced with coexistence with environment of nanomaterials and heavy metals.

## 1. Introduction

Nanomaterials (NMs) with a series of superior performances have been widely used in medicine, mechanics, electronics, agriculture and the energy field [1,2,3,4]. As one of the most attractive NMs with multiple forms, carbon-based nanomaterials (CBNs) with nano-sized and unique physico-chemical properties, including graphene oxide (GO), single-walled carbon nanotubes, multiple-walled carbon nanotubes, carbon nanoparticles and fullerenes, provoke tremendous scientific interest for nanotechnology development of a wide range of medical and industrial applications [5,6,7,8,9,10,11,12,13,14]. Among them, GO, as a novel CBN, presents the widest application duo to more fascinating properties, such as high surface area, good thermal stability, excellent mechanical strength, high optical transmittance and electrical conductivity [15,16,17,18]. As a chemically modified graphene, GO contains a single-atom-thick two-dimensional sheet of carbon atoms [19,20]. GO carries large numbers of oxygen-containing functional groups such as alcohols, epoxides and carboxylic acids, and thus has exceptional structure characteristics, in which numerous hydrophobic sp(2) clusters are isolated within the hydrophilic sp(3) C-O matrix [21,22,23]. Because of those unique composite properties, GO has been widely applied to every respect of social life especially in the biomedical field such as drug delivery, biosensors, antibiotics, vaccine enhancers, photo-thermal therapy and cancer treatments [24,25,26,27,28,29]. By 2020, the market for GO products is approaching $675 million, which may result in a large number of graphene-based waste [30]. The intense demand and broad application of GO will lead to a significant increase in the amount of GO released into the environment, which may further bring unpredictable effects on human health, organism and ecology security [31]. Therefore, it is a critical issue for clarifying the potential effect of GO on organism.

Currently, numerous reports have documented the effects of GO on bacteria, animals and humans [32,33,34,35,36,37], and a few studies concern the effect of GO on crop plants. For instance, Begum et al. [38] reported that, under hydroponic conditions, a large number of GO accumulated on the roots of cabbage, tomato and red spinach which eventually resulted in the growth inhibition of these plants. A similar phenomenon was also found by Jiao et al. [39], which showed that GO in solution culture could significantly cause stress damage of plant cells and decrease the length of tobacco roots. Cheng et al. [40] showed that GO could lead to a decrease in the fresh root weight in *Brassica napus* L. In contrast, Hu et al. [41] reported that 0.1–10 mg L^−1^ GO did not significantly inhibit the root and shoot length of wheat (*Triticum aestivum* L.) compared with the control. He et al. [23] found that GO could stimulate the growth of plant, and low-concentration GO significantly promoted the germination of spinach and chive. Gao et al. [42] investigated that low-concentration GO exhibited a limited toxic effect on wheat and 5–40 mg L^−1^ GO stimulated a significant increase in the Cd content in the plant. A previous study showed that 5 mg L^−1^ GO had a promoting effect on the fresh weight of root and overground part of rice seedlings and 50 mg L^−1^ GO had an inhibitive effect on the fresh weight of the overground part of the plant [43]. Jiao et al. [44] observed that 20 mg L^−1^ GO promoted tomato root growth. Guo et al. [45] also showed that 50 and 100 mg L^−1^ GO enhanced the root development and increased biomass accumulation of mature tomato plants, and 200 mg L^−1^ GO did not significantly affect plant growth. This difference above may be related to plant species, GO concentrations and exposure duration [46]. However, these results above were based on limited research and lack of better knowledge about more plant species. Greening plants are important living components of the urban ecosystem, while few studies on GO involved greening plants; therefore, it was very necessary to assess the effect of different concentrations of GO on the greening plants.

In urban environments, pollutants cannot exist in isolation. In addition to the potential impact of GO, excessive heavy metals derived from the metallurgy and mining industry, exhaust emission and sewage irrigation were released into the urban environment and pose a serious threat to the survival of human beings and greening plants [47,48,49,50,51]. Cadmium (Cd), as one of the most toxic heavy metals, has become a global issue of common concern due to its high persistency, strong water solubility and carcinogenicity [52,53,54]. The content of Cd in soil exceeds the safety standard of the national soil quality, which will not only result in the inhibition of the eco-physiological characteristics including plant growth, photosynthesis and carbon sequestration and oxygen release functions, but also bring threats to human health induced by food chain transmission [55,56,57,58]. In this case, it is an urgent necessity to resolve the Cd contamination in the urban environment. Phytoremediation is an emerging technology of contamination control, which takes advantage of a hyperaccumulator to clean up hazardous heavy metals in environment [59,60,61]. Meanwhile, GO is considered as an efficient adsorbent CBNs applied to remove heavy metals from aquatic environment or wastewater due to its excellent physicochemical properties, such as strong hydrophilia, abundant oxygen functional groups, and large specific surface area [9,11,12,20,30,62,63,64,65]. It is obvious to show that greening hyperaccumulator and GO are both beneficial for removing heavy metals in contaminated urban environment; however, little information is available on the effects of GO on a greening hyperaccumulator under heavy-metal Cd stress.

*Lonicera japonica* Thunb. is a popular greening plant in urban areas and has various excellent characteristics such as easy cultivation, high biomass and strong resistance [66]. Our previous studies reported that *L. japonica* can accumulate cadmium (Cd) in stems and shoots above 100 μg g^−1^ dry tissue [67], which is the threshold value of a Cd-hyperaccumulator, and it is considered as a new woody Cd-hyperaccumulator [68,69,70,71,72,73,74]. In the present study, *L. japonica,* as a typical greening hyperaccumulator, is chosen to clarify the effect of GO on Cd accumulation and eco-physiological characteristics (including the growth, photosynthetic parameters, carbon sequestration and oxygen release functions) of the plant under Cd stress. It will be helpful to acquire a better understanding with the eco-physiological mechanisms of greening plants faced with an environment of nanomaterials and heavy metals, and confirm the phytoremediation potential of a woody greening hyperaccumulator regulated by a novel CBNs (GO).

## 2. Materials and Methods

### 2.1. Chemicals

The GO used in the study is a GO aqueous dispersion (5 mg mL^−1^, single-layer GO content > 98%, particle size 0.5–5 μm) purchased from Jiangsu Xianfeng Nano Material Technology Co., Ltd. (Nanjing, China), which has the characteristics of good water solubility, low impurities and strong stability. It was prepared using the improved Hummers method, and then subjected to ultrasonic treatment in deionized water to obtain a stable GO aqueous dispersion. CdCl_2_·2.5H_2_O (analytically pure, >99%) and all other chemicals were obtained from Kermel Chemical Reagent Co., Ltd. (Tianjin, China).

### 2.2. Materials and Treatments

Plant materials (*Lonicera japonica* Thunb.) in the study were derived from a non-contaminated field of Shenyang Agricultural University and propagated in sterilized sand. After 8 weeks, uniform plants were transformed to 500 mL adumbral containers with nutrient medium, 4 plants for each container, placed in an artificial climate chamber under controlled conditions: 16 h light/8 h dark cycle, 800–1000 μmol m^−2^ s^−1^ photosynthetic photo flux density (PPFD), with a 25 °C/18 °C day/night temperature and a relative humidity of 60%. The nutrient medium was the modified Hoagland solution containing the following ingredients (mmol L^−1^): Ca(NO_3_)_2_ × 4H_2_O 5.00, MgSO_4_ × 7H_2_O 2.00, KNO_3_ 5.00, KH_2_PO_4_ 1.00, H_3_BO_3_ 0.05, ZnSO_4_ × 7H_2_O 0.80 × 10^−3^, MnCl_2_ × 4H_2_O 9.00 × 10^−3^, CuSO_4_ × 5H_2_O 0.30 × 10^−3^, (NH_4_)_6_Mo_7_O_24_ × 4H_2_O 0.02 × 10^−3^, Fe-EDTA 0.10. The pH of each solution was adjusted daily to 5.8 ± 0.1 with 0.1 M HCl or 0.1 M NaOH, and the nutrient medium in each container was renewed once every 3 days. Different concentrations of Cd^2+^ (0, 5 and 25 mg L^−1^) were obtained by CdCl_2_·2.5H_2_O solution. According to the reported ranges of concentrations in previous studies [49,50,51], the experiment sets up four GO treatments, which contain 0, 10, 50 and 100 mg L^−1^, respectively. The combined concentrations of GO and Cd (GO-Cd) were prepared by mixing GO and Cd alone, and twelve GO-Cd treatments were set up in the study as follows in Table 1. Four replications were applied in each GO-Cd treatment experiment. The seedlings of *L. japonica* were collected for analysis 4 weeks after GO-Cd treatment initiation.

### 2.3. Determination of Plant Biomass and Cd Content

The harvested plants were rinsed with tap water, and then separated into root and shoots. These plant tissues were separately rinsed with distilled water and finally with deionized water, wiped for surface moisture and weighed. These plant tissues were dried at 105 °C for 20 min, then they were dried at 70 °C until the weight became constant [72].

Dried plant tissues were weighed and ground to fine powder. These fine powders were digested with a concentrated acid mixture of HNO_3_/HClO_4_ (3:1, *v*/*v*) in a microwave digestion instrument (MARS5, CEM, Matthews, NC, USA). The Cd contents in the plant tissues (root and shoots) were determined by a flame atomic absorption spectrometer (AAS 3110 Perkin-Elmer, Waltham, MA, USA).

### 2.4. Assays of Photosynthetic Parameters

As one of the most important photosynthetic parameters, the net photosynthetic rate (P_n_) of the plant was measured by a portable LI-6400 photosynthesis system (Lincoln, NE, USA) under different GO-Cd treatments. During the process of GO-Cd treatments, these parameters including light level, CO_2_ concentration and leaf temperature inside the leaf chamber were kept stable at 1000 μmol m^−2^ s^−1^ PPFD, 25 ± 0.3 °C and 380 ± 5 μmol CO_2_ mol^−1^.

### 2.5. Analysis of Carbon Sequestration and Oxygen Release Functions

The functions of carbon sequestration and oxygen release were measured according to the previous research [75]. The values of carbon sequestration and oxygen release values were decided by diurnal assimilation amounts (*P*) that can be calculated as Equation (1):

(1) $$P=\sum_{i=1}^{j}\left[(p_{i+1}+p_{i})÷2\times (t_{i+1}-t_{i})\times 3600÷1000\right]$$

where *P* is the diurnal assimilation amount (mmol m^−2^ s^−1^), *p_i_* is the instantaneous photosynthetic rate at the initial measurement point (μmol m^−2^ s^−1^), *p_i+_*_1_ is the instantaneous photosynthetic rate of the next measurement point (μmol m^−2^ s^−1^), *t_i_* is the instantaneous time of the initial measurement point (h), *t_i_*_+1_ is the instantaneous time of the next measurement point (h), *j* is test times, 3600 refers to 3600 s per hour, and 1000 refers to 1000 μmol per mmol.

Carbon sequestration per unit leaf area (W_CO_2__) and oxygen release per unit leaf area (W_O_2__) of the plant can be calculated as Equations (2) and (3):

W_CO_2__ *= P* × 44/1000 (2)

W_O_2__ = *P* × 32/1000 (3)

where W_CO_2__ is carbon sequestration per unit leaf area (g m^−2^ d^−1^), 44 is the molar mass of CO_2_; W_O2_ is oxygen release per unit leaf area (g m^−2^ d^−1^), 32 is the molar mass of O_2_.

### 2.6. Statistical Analyses

The data in the study are presented as means ± SD (standard deviation). SPSS 22.0 (SPSS Inc., Chicago, IL, USA) and Microsoft Office Excel 2020 (Microsoft Corporation, Redmond, WA, USA) were applied for statistical analysis. The significant differences between the GO-Cd treatments were presented at the *p* < 0.01 and *p* < 0.05 levels.

## 3. Results and Discussion

### 3.1. Cd Accumulation of L. japonica

**Figure 1:**
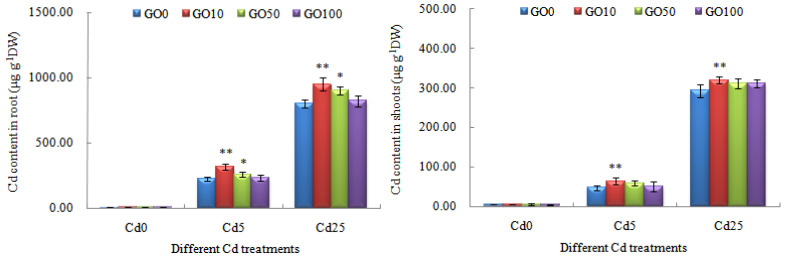
The effect of different GO-Cd treatments on Cd contents in root and shoots of *L. japonica*. The “**” indicates a significant difference compared with the control (*p* < 0.01), and the “*” indicates a significant difference compared with the control (*p* < 0.05). Mean ± SD.

After 4-week GO-Cd treatments, Cd contents in root and shoots of *L. japonica* are shown in Figure 1. With the increase in Cd concentration in solution, Cd contents in roots and shoots of *L. japonica* all increased significantly compared to the control, which is consistent with our previous research findings [69,70,74]. Under 5 and 25 mg L^−1^ Cd stress without GO treatments, the Cd contents in the root of the plant were 223.75 ± 17.23 μg g^−1^ (GO0-Cd5) and 800.25 ± 31.84 μg g^−1^ DW (GO0-Cd25). Under GO treatments, the Cd contents in the root of the plant had an increasing trend compared with the control, especially exposed to high concentrations (10 and 50 mg L^−1^) GO in solution. Under 5 and 25 mg L^−1^ Cd stress, the Cd contents in the root of the plant were enhanced significantly by 10 mg L^−1^ GO treatment (*p* < 0.01), which reached 318.13 ± 22.05 μg g^−1^ DW (GO10-Cd5) and 949.67 ± 52.14 μg g^−1^ DW (GO10-Cd25), respectively. Similar results were reported by Yin et al. [76], who found that 100 to 500 mg L^−1^ GO promoted the increased Cd contents in roots of maize exposed to 20 mg L^−1^ Cd, possibly due to Cd^2+^ in roots more adsorbed through adhering GO particles. When the GO concentration in solution was added to 50 mg L^−1^ (exposed to 5 and 25 mg L^−1^ Cd), the Cd contents in the root of the plant increased significantly (*p* < 0.05), which reached 257.38 ± 18.10 μg g^−1^ DW (GO50-Cd5) and 903.41 ± 32.29 μg g^−1^ DW (GO50-Cd25), raising by 15.03% and 12.89% compared with the GO0-Cd5 and GO0-Cd25 treatments. Our research results are in agreement with the results of the study by Gao et al. [42], who showed that when GO was supplied to wheat in root tissues, low-concentration GO promoted a significant increase in Cd content in wheat. With the increase in GO concentration in solution, the Cd contents in root of the plant had no significant increase compared with the GO0-Cd5 (T_2_) and GO0-Cd25 (T_3_) treatments. The reason might be that the limited root surface area cause the root to not be able to adhere more GO particles, or that the carboxylate groups at GO edges provide active sites to accumulate Cd^2+^ in the interlayer space and reduce root uptake gradually [76,77].

By comparison, the Cd contents in shoots of *L. japonica* had a similar change trend with the Cd contents in root under different GO-Cd treatments. Under 5 and 25 mg L^−1^ Cd stress, the Cd contents in shoots of the plant were enhanced significantly by 10 mg/L GO treatment (*p* < 0.01), which reached 63.42 ± 8.55 μg g^−1^ DW (GO10-Cd5) and 320.03.67 ± 9.47 μg g^−1^ DW (GO10-Cd25), respectively. When the GO concentration in solution was added to 50 mg L^−1^ (exposed to 5 and 25 mg L^−1^ Cd), the Cd contents in shoots of the plant had an increased trend, which reached 58.36 ± 5.94 μg g^−1^ DW (GO50-Cd5) and 311.29 ± 13.51 μg g^−1^ DW (GO50-Cd25), raising by 23.96% and 6.21% compared with the GO0-Cd5 and GO0-Cd25 treatments. With the increase in GO concentration in solution, the Cd contents in shoots of the plant had no significant increase compared with the GO0-Cd5 (T_2_) and GO0-Cd25 (T_3_) treatments. This is may be due to the reason that GO can directly and indirectly regulate the uptake of heavy metals and the regulation effect was concentration-dependent [41]. The maximum rates of Cd accumulation in roots and shoots of the plant increased by 10 mg L^−1^ GO (exposed to 5 mg L^−1^ Cd) were 42.18% and 34.71% compared with the GO0-Cd5 (T_2_) treatment, indicating that low-concentration GO (10 mg L^−1^) combined with low-concentration Cd (5 mg L^−1^) might stimulate the absorption of Cd by *L. japonica*, which could be applied on a suitable concentration GO-enhanced phytoremediation of *L. japonica* under a Cd-contaminated environment. The suitable GO concentration is important for improving phytoremediation ability. This is possibly because suitable concentration GO can positively modulate the metabolic processes and adsorption mechanisms in *L. japonica* including the improved eco-physiological responses for maintaining good tolerance, the stimulated activity of antioxidant enzymes to reduce excessive reactive oxygen species (ROS) production, and the promoted conversion of exchangeable Cd^2+^ which is more available for plant absorption. The specific mechanism still needs further exploration. Positive effects of GO on Cd accumulation in duckweed were also observed by Yang et al. [78]. The possible reasons for this are the large specific surface area and adsorption capacity of GO, co-transport of GO and heavy metal, and regulated transporter gene expression [79,80,81].

### 3.2. Growth Charateristics of L. japonica

**Figure 2:**
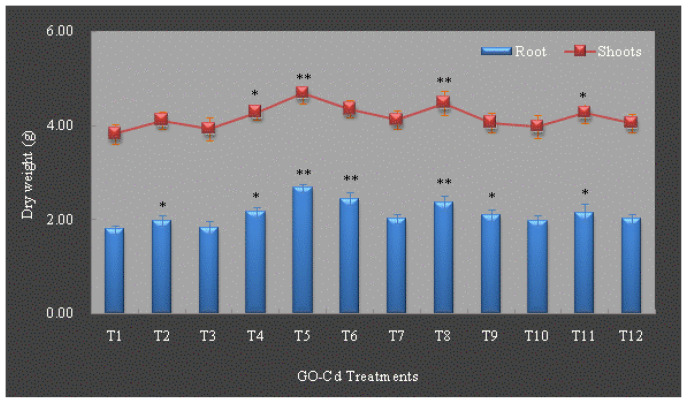
The effect of different GO-Cd treatments on root and shoots biomass dry weight (g) of *L. japonica*. The “**” indicates a significant difference compared with the control (*p* < 0.01), and the “*” indicates a significant difference compared with the control (*p* < 0.05). Mean ± SD.

Several studies found that growth indicators are a sensitive parameter when the plants are subject to external environmental stress [82,83]. After 4-week GO-Cd treatments, the growth characteristics in terms of root and shoot biomass dry weight in *L. japonica* are shown in Figure 2. Under T_1_–T_3_ treatments (under Cd stress without GO treatments), the dry weight of root biomass increased significantly exposed to 5 mg L^−1^ Cd (*p* < 0.05), then had a decreased trend exposed to 25 mg L^−1^ Cd and had no significant change compared with the control, indicating that low-concentration Cd could promote the growth of the plant. The results are in accordance with our previous studies, which observed an inverted U-shaped dose–response curve of the growth characteristics under different concentrations of Cd treatments, indicating that the hormetic effect of low-concentration Cd occurred in *L*. *japonica* [68,69,70,71,72,84]. The phenomenon of hormesis has also been found in barley [85]. Under GO treatments without Cd stress (T_4_, T_7_ and T_10_), the dry weight of root biomass showed an increase, especially under 10 mg L^−1^ GO (T_4_), 20.67% higher than the control (T_1_, GO0-Cd0), which may due to the reason that the GO with various oxygen-containing functional groups collected water, and the hydrophobic sp(2) domains of GO transported water to the roots to accelerate the growth of plants [23]. Under GO treatments and Cd stress (T_5_, T_6_, T_8_, T_9_, T_11_ and T_12_), different concentrations of GO increased the dry weight of root biomass exposed to different concentrations of Cd, especially under 10 mg L^−1^ GO; the dry weight of root biomass exposed to 5 mg L^−1^ Cd (T_5_) and 25 mg L^−1^ Cd (T_6_) were enhanced significantly by 34.85% and 33.33% higher than the GO0-Cd5 (T_2_) and GO0-Cd25 (T_3_) treatments, which showed that the cooperation of low-concentration GO (10 mg L^−1^) and low-concentration Cd (5 mg L^−1^) could significantly promote the growth of root, and with the increase in Cd stress, 10 mg L^−1^ GO could alleviate the toxic effect of Cd on the root of the plant. A similar phenomenon was also observed by He et al. [77], who found that the seedlings of the rice (*Oryza sativa L. ssp. japonica*) showed the largest fresh weight value under Cd and GO treatments. The reason might be that low-concentration GO is relatively biocompatible [86,87] and could provide water and enhance nutrients for plant growth within limits [23,88]. Higher-concentration GO could affect the permeability of cell membranes and metabolic processes of plants [78].

In contrast to root, the dry weight of shoot biomass of *L. japonica* had a similar change trend under different GO-Cd treatments. Under T_1_–T_3_ treatments (under Cd stress without GO treatments), the dry weight of shoot biomass showed an inverted U-shaped dose–response curve, indicating that the low-concentration Cd had a hormetic effect on the growth of shoots. Under GO treatments without Cd stress (T_4_, T_7_ and T_10_), the dry weight of shoot biomass showed an increase, especially under 10 mg L^−1^ GO (T_4_), 12.04% higher than the control (T_1_, GO0-Cd0). A similar phenomenon was also reported by other researchers when the plants were subjected to different environmental stresses, which is considered as hormesis that may have resulted from the overcompensating behavior of the organism responding to the disrupted homeostasis [89,90,91,92,93,94]. Under GO treatments and Cd stress (T_5_, T_6_, T_8_, T_9_, T_11_ and T_12_), different concentrations of GO increased the dry weight of shoot biomass exposed to different concentrations of Cd, especially under 10 mg L^−1^ GO; the dry weight of shoots biomass exposed to 5 mg L^−1^ Cd (T_5_) were enhanced significantly and reached 4.69 ± 0.22 μg g^−1^ DW, increasing by 14.11% compared with the GO0-Cd5 (T_2_) treatment, which showed that low-concentration GO (10 mg L^−1^) combined with low-concentration Cd (5 mg L^−1^) could significantly stimulate the growth of shoots. With the increase in GO concentration in solution, the dry weight of root and shoot biomass had no significant increase compared with the T_1_–T_3_ treatments (under Cd stress without GO treatments), which indicated that GO has a limited concentration effect on alleviating the toxic effect of Cd, and the enhanced accumulation of Cd in roots and shoots stimulated by GO might gradually lead to the increase in cytotoxicity. The results are consistent with the findings of Hu et al. [41] and Yin et al. [76], who proposed that the effects of GO on plant growth depend on GO concentration.

### 3.3. Photosynthetic Parameters of L. japonica

**Figure 3:**
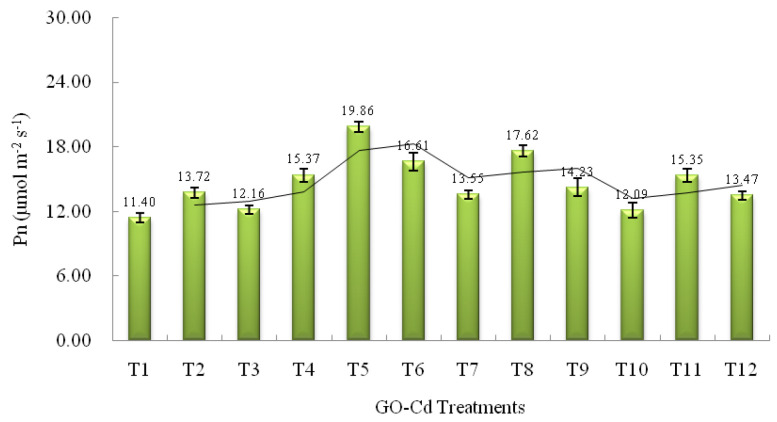
The effect of different GO-Cd treatments on net photosynthesis rate (P_n_) in *L. japonica*. Mean ± SD.

Photosynthesis of plants is the source of the material and energy required by most organisms on earth; however, as a sensitive site for environmental factors, photosynthesis is susceptible to external environmental changes [95]. It is reported that the net photosynthesis rate (P_n_) is usually used as an important indicator for measuring the ability of light energy utilization [96]. The effects of different GO-Cd treatments on Pn in *L. japonica* are shown in Figure 3. When the plant was exposed to 5 mg L^−1^ Cd stress without GO treatments (T_2_), the value of P_n_ in *L. japonica* increased significantly and reached 13.72 ± 0.51 μmol m^−2^ s^−1^, which is 20% compared with the control. With the increase in Cd stress, the value of P_n_ in *L. japonica* had a decreased trend and had no significant increase compared with the control, indicating that low-concentration Cd could stimulate the photosynthesis of the plant. The results are in agreement with the growth responses of *L. japonica*, which showed an inverted U-shaped dose–response curve under different concentrations of Cd treatments. Yet, several researchers found that different plants exhibit different response characteristics under GO treatments. Chen et al. [97] reported that GO had serious toxicity and induced the inhibition of photosynthesis in naked oats (*Avena sativa* L.). Zhao et al. [98] showed that GO exposure had adverse effects on the photosynthetic parameters of white clover (*Trifolium repens* L.), the reason for which may be a decreased metabolism, as shown by electron transport system activity [99]. Hazeem et al. [100] found that low concentrations of GO exposure can improve photosynthetic pigment content of the marine alga *Picochlorum* sp., and higher concentrations of GO exposure had a negative effect on the photosynthetic pigment content of the plant. However, in the present study, under GO treatments without Cd stress (T_4_, T_7_ and T_10_), the value of P_n_ in *L. japonica* showed an increasing trend, especially under 10 mg L^−1^ GO (T_4_), the value of P_n_ in the plant increased significantly, 35% higher than the control (T_1_, GO0-Cd0). The P_n_ value in *L. japonica* had an increase of 19% compared with the control when the plant was exposed to 50 mg L^−1^ GO (T_7_) treatment. The significant increase in P_n_ when *L. japonica* was exposed to low concentrations of GO treatments may stem from a stimulating effect by hormesis. A similar result was also found by Zhou et al. [101], which showed that GO exposure can promote photosynthesis performance of *Iris pseudacorus*. The reasons for this might result from optimizing the electron transport at the acceptor side of PSII and improving the energy conversion efficiency of PSII. When GO concentration in solution was up to 100 mg L^−1^, the value of P_n_ in *L. japonica* showed a slight increase of 6% and had no significant increase compared with the control, indicating that the plant still kept a good ability of light energy utilization even under high concentrations of GO treatment. Under GO treatments and Cd stress (T_5_, T_6_, T_8_, T_9_, T_11_ and T_12_), different concentrations of GO increased the value of P_n_ in *L. japonica* exposed to different concentrations Cd, especially under 10 mg L^−1^ GO, where the P_n_ value in *L. japonica* exposed to 5 mg L^−1^ Cd (T_5_) was enhanced significantly and reached 19.86 ± 0.42 μg g^−1^ DW, increasing by 45% compared with the GO0-Cd5 (T_2_) treatment, which showed that the cooperation of low-concentration GO (10 mg L^−1^) and low-concentration Cd (5 mg L^−1^) has dual hormetic effect on the photosynthesis of the plant. With the increase in GO and Cd concentrations in solution, the P_n_ value in *L. japonica* still kept an increase, indicating the plant did not suffer GO or metal toxicity and generated a product of metabolism for metal absorption, plant growth and photosynthesis, which might be related with the strong tolerance and hyperaccumulation characteristics of *L. japonica* combined with environmental stress [70,102].

### 3.4. Carbon Sequestration and Oxygen Release Functions of L. japonica

It is well known that carbon sequestration and oxygen release functions are considered as crucial parameters to assess the ability of plant photosynthesis [103]. To investigate carbon sequestration and oxygen release functions, it is helpful to explore the ecological service function of the greening plants [104]. The effect of different GO-Cd treatments on the carbon sequestration and oxygen release characteristics of *L. japonica* are shown in Table 2. In the present study, when *L. japonica* was exposed to 5 mg L^−1^ Cd stress without GO treatments (T_2_), the carbon sequestration per unit leaf area and oxygen release per unit leaf area of the plant had significant increases and reached 12.06 ± 0.31 g m^−2^ d^−1^, 8.77 ± 0.28 g m^−2^ d^−1^, respectively, which is 19.05% compared with the control. The results indicated that low-concentration Cd could stimulate the carbon sequestration and oxygen release functions of the plant, which is in agreement with the response of photosynthetic parameters in terms of increased P_n_ value. When Cd treatment concentration was up to 25 mg L^−1^, the carbon sequestration per unit leaf area and oxygen release per unit leaf area of *L. japonica* showed a slight increase of 11.35% and had no significant increase compared with the control. Under GO treatments without Cd stress (T_4_, T_7_ and T_10_), the carbon sequestration per unit leaf area and oxygen release per unit leaf area of *L. japonica* showed an increase, especially under 10 mg L^−1^ GO (T_4_), 28.73% higher than the control (T_1_, GO0-Cd0). When *L. japonica* was exposed to 10 mg L^−1^ GO treatment, the carbon sequestration per unit leaf area and oxygen release per unit leaf area of the plant had significant increases and reached 13.04 ± 0.53 g m^−2^ d^−1^ and 9.48 ± 0.21 g m^−2^ d^−1^, respectively. Under low concentrations of GO treatments, the significant increase in carbon sequestration and oxygen release values of *L. japonica* may result from the stimulated photosynthesis by hormesis effect, which is represented by the change in P_N_ value. This is identical to the relevant study, which showed that main biological components required for carbon sequestration and oxygen release all source from the process of plant photosynthesis [105]. It was also reported that the greening plants with functional carbon sequestration and oxygen release characteristics could exchange more CO_2_ and O_2_ with the environmental medium, then change more light energy into nutrients stored in plants [106]. When GO treatment concentration was up to 100 mg L^−1^, the carbon sequestration per unit leaf area and oxygen release per unit leaf area of the plant had significant increases and reached 11.25 ± 0.29 g m^−2^ d^−1^ and 8.18 ± 0.36 g m^−2^ d^−1^, respectively, which is 11.06% compared with the control, indicating that the plant still had good functions of carbon sequestration and oxygen release even under high concentrations of GO treatment. Under GO treatments and Cd stress (T_5_, T_6_, T_8_, T_9_, T_11_ and T_12_), different concentrations of GO increased the carbon sequestration per unit leaf area and oxygen release per unit leaf area of the plant exposed to different concentrations of Cd, especially under 10 mg L^−1^ GO, where the carbon sequestration per unit leaf area and oxygen release per unit leaf area of the plant exposed to 5 mg L^−1^ Cd (T_5_) were enhanced significantly by 38.89% higher than the GO0-Cd5 (T_2_) treatment, which showed that the cooperation of low-concentration GO (10 mg L^−1^) and low-concentration Cd (5 mg L^−1^) could significantly stimulate the carbon sequestration and oxygen release functions of the plant (*p* < 0.01). It is shown that the carbon sequestration per unit leaf area could predict the carbon sequestration capacity of plants through their photosynthesis, and therefore it was directly used for the evaluation of the carbon sequestration functions in different types of plants [103,107]. In the present study, *L. japonica* still kept an increase by 20.34% compared to the control even under 100 mg L^−1^ GO and 25 mg L^−1^ Cd (T_12_) treatments, which indicated that the plant had a strong capacity of carbon sequestration under the mutual influence of environmental stress.

## 4. Conclusions

In the present study, the effect of GO on Cd accumulation and eco-physiological characteristics (including the growth, photosynthetic parameters, carbon sequestration and oxygen release functions) of *L. japonica* under different concentrations of Cd treatments were investigated. It is observed that the suitable GO concentration is important for improving phytoremediation ability. Low-concentration GO (10 mg L^−1^) combined with low-concentration Cd (5 mg L^−1^) might stimulate the absorption of Cd by *L*. *japonica*, which could be applied on a suitable concentration GO-enhanced phytoremediation of *L*. *japonica* under a Cd-contaminated environment. Under GO-Cd treatments, it is shown that the cooperation of low-concentration GO (10 mg L^−1^) and low-concentration Cd (5 mg L^−1^) could significantly stimulate the growth, photosynthesis, carbon sequestration and oxygen release functions of the plant. With the increase in treatment concentrations, *L. japonica* still kept an increase of 20.34% compared to the control even under 100 mg L^−1^ GO and 25 mg L^−1^ Cd treatments, which indicated that the plant had a strong capacity for carbon sequestration under the mutual influence of environmental stress. In our previous studies, it was investigated that *L. japonica* is a greening Cd-hyperaccumulator with dual merits of phytoremediation and decoration, which will bring social and environmental benefits on urban construction. Therefore, we will consider these good capacities of carbon sequestration and hyperaccumulation comprehensively by combing practical applications, which could provide a more reasonable suggestion for city’s policymakers. Otherwise, the study will supply referable achievement for the ecological responses of the greening plants to combined environmental stress.

## Figures and Tables

**Table 1:** Different experimental treatments.

Test Number	GO-Cd Treatments	GO Concentration in Solution (mg L^−1^)	Cd Concentration in Solution (mg L^−1^)
T_1_	GO0-Cd0	0	0
T_2_	GO0-Cd5	0	5
T_3_	GO0-Cd25	0	25
T_4_	GO10-Cd0	10	0
T_5_	GO10-Cd5	10	5
T_6_	GO10-Cd25	10	25
T_7_	GO50-Cd0	50	0
T_8_	GO50-Cd5	50	5
T_9_	GO50-Cd25	50	25
T_10_	GO100-Cd0	100	0
T_11_	GO100-Cd5	100	5
T_12_	GO100-Cd25	100	25

**Table 2:** The effect of different GO-Cd treatments on the carbon sequestration and oxygen release characteristics of *L. japonica*.

Test Number	GO-Cd Treatments	Carbon Sequestration Per Unit Leaf Area (g m^−2^ d^−1^)	Oxygen Release Per Unit Leaf Area (g m^−2^ d^−1^)
T_1_	GO0-Cd0	10.13 ± 0.26	7.37 ± 0.14
T_2_	GO0-Cd5	12.06 ± 0.31 *	8.77 ± 0.28 *
T_3_	GO0-Cd25	11.28 ± 0.25	8.20 ± 0.33
T_4_	GO10-Cd0	13.04 ± 0.53 **	9.48 ± 0.21 **
T_5_	GO10-Cd5	16.75 ± 0.42 **	12.18 ± 0.35 **
T_6_	GO10-Cd25	14.62 ± 0.17	10.63 ± 0.29
T_7_	GO50-Cd0	12.39 ± 0.31 *	9.01 ± 0.50 *
T_8_	GO50-Cd5	15.17 ± 0.16 **	11.03 ± 0.41 **
T_9_	GO50-Cd25	13.41 ± 0.37	9.75 ± 0.18
T_10_	GO100-Cd0	11.25 ± 0.29	8.18 ± 0.36
T_11_	GO100-Cd5	14.08 ± 0.33 *	10.24 ± 0.25 *
T_12_	GO100-Cd25	12.19 ± 0.24	8.87 ± 0.19

The “**” indicates a significant difference compared with the control (*p* < 0.01), and the “*” indicates a significant difference compared with the control (*p* < 0.05). Mean ± SD.

## Data Availability

Data are contained within the article.
